# Artificial Intelligence in Adult Cardiovascular Medicine and Surgery: Real-World Deployments and Outcomes

**DOI:** 10.3390/jpm16020069

**Published:** 2026-01-30

**Authors:** Dimitrios E. Magouliotis, Noah Sicouri, Laura Ramlawi, Massimo Baudo, Vasiliki Androutsopoulou, Serge Sicouri

**Affiliations:** 1 Department of Cardiac Surgery Research, Lankenau Institute for Medical Research, Wynnewood, PA 19096, USA; magouliotisd@mlhs.org (D.E.M.); laura.ramlawi@gmail.com (L.R.); massimo.baudo@icloud.com (M.B.); 2 Department of Neuroscience, University of Pittsburgh, Pittsburgh, PA 15260, USA; nps67@pitt.edu; 3 Department of Cardiothoracic Surgery, University of Thessaly, Biopolis, 41110 Larissa, Greece; androutsopoulouvasiliki@uth.gr

**Keywords:** artificial intelligence, machine learning, cardiac surgery, computer vision

## Abstract

Artificial intelligence (AI) is rapidly reshaping adult cardiac surgery, enabling more accurate diagnostics, personalized risk assessment, advanced surgical planning, and proactive postoperative care. Preoperatively, deep-learning interpretation of ECGs, automated CT/MRI segmentation, and video-based echocardiography improve early disease detection and refine risk stratification beyond conventional tools such as EuroSCORE II and the STS calculator. AI-driven 3D reconstruction, virtual simulation, and augmented-reality platforms enhance planning for structural heart and aortic procedures by optimizing device selection and anticipating complications. Intraoperatively, AI augments robotic precision, stabilizes instrument motion, identifies anatomy through computer vision, and predicts hemodynamic instability via real-time waveform analytics. Integration of the Hypotension Prediction Index into perioperative pathways has already demonstrated reductions in ventilation duration and improved hemodynamic control. Postoperatively, machine-learning early-warning systems and physiologic waveform models predict acute kidney injury, low-cardiac-output syndrome, respiratory failure, and sepsis hours before clinical deterioration, while emerging closed-loop control and remote monitoring tools extend individualized management into the recovery phase. Despite these advances, current evidence is limited by retrospective study designs, heterogeneous datasets, variable transparency, and regulatory and workflow barriers. Nonetheless, rapid progress in multimodal foundation models, digital twins, hybrid OR ecosystems, and semi-autonomous robotics signals a transition toward increasingly precise, predictive, and personalized cardiac surgical care. With rigorous validation and thoughtful implementation, AI has the potential to substantially improve safety, decision-making, and outcomes across the entire cardiac surgical continuum.

## 1. Introduction

Artificial intelligence (AI) is rapidly reshaping the landscape of cardiovascular medicine, offering new tools to enhance diagnosis, prognostication, and clinical decision-making. In adult cardiac surgery, where perioperative risk is substantial and outcomes depend on complex, data-driven judgments, AI has emerged as a transformative force capable of augmenting surgeon performance and system efficiency. Traditional risk-prediction models such as EuroSCORE II and the STS risk calculators, although widely adopted, are constrained by linear assumptions and limited variable interactions, often underperforming in heterogeneous contemporary cohorts [1,2]. In contrast, machine learning (ML) and deep learning (DL) algorithms can process high-dimensional datasets, account for nonlinear relationships, and generate individualized risk estimates with superior accuracy [3].

Over the past decade, AI applications in cardiac surgery have transitioned from experimental modeling to real-world deployment. ML-based mortality and morbidity prediction tools have demonstrated improved calibration and discrimination across diverse populations, including patients undergoing coronary artery bypass grafting (CABG), aortic surgery, and complex valve interventions [4]. Natural language processing (NLP) systems are increasingly used to extract granular clinical variables from electronic health records (EHRs), automating data collection and enhancing quality improvement initiatives [5]. Similarly, computer vision algorithms have enabled automated assessment of operative videos, workflow recognition, and intraoperative technical performance, setting the foundation for future AI-assisted operating rooms [6].

Beyond prediction and automation, AI-driven optimization strategies have shown measurable improvements in perioperative outcomes. Real-world deployments include ML-powered early warning systems for hemodynamic instability, postoperative decompensation, and intensive care unit (ICU) deterioration, resulting in earlier intervention and reduced failure-to-rescue rates [7]. AI-based scheduling and resource-allocation platforms have increased operating room (OR) efficiency and reduced surgical delays in high-volume centers [8]. Importantly, several health systems have integrated these technologies on a large scale, demonstrating not only feasibility but also measurable reductions in mortality, complications, and length of stay, signaling that AI’s impact is no longer theoretical but demonstrably clinical [9].

Despite these advances, challenges remain related to model transparency, data quality, algorithmic bias, and regulatory acceptance. Nevertheless, the rapid adoption of AI across leading cardiovascular centers reflects a paradigm shift toward data-enriched surgical care. This review synthesizes real-world deployments of AI in adult cardiac surgery and evaluates their clinical impact, emphasizing opportunities for scalable, safe, and outcome-driven integration.

## 2. Literature Search Strategy and Study Selection

This narrative review was conducted to synthesize evidence on real-world applications of artificial intelligence in adult cardiovascular medicine and cardiac surgery, with an emphasis on clinically implemented or implementation-adjacent systems. A structured literature search was performed in PubMed/MEDLINE, Scopus, and Web of Science, covering studies published between January 2015 and October 2025, reflecting the modern era of machine learning and deep learning in clinical practice.

Although this review focuses on adult cardiac surgery, relevant evidence from adjacent domains (including general cardiology, thoracic surgery, and critical care) is discussed where direct cardiac-surgical data remain limited. In such cases, these examples are presented explicitly as extrapolative or translational evidence, intended to inform potential applicability rather than to imply established efficacy within cardiac surgery itself. Whenever possible, cardiac-surgery–specific studies are prioritized, and extrapolated findings are clearly framed as hypothesis-generating rather than definitive.

Search terms included combinations of “artificial intelligence,” “machine learning,” “deep learning,” “cardiac surgery,” “cardiovascular surgery,” “perioperative,” “intensive care,” “risk prediction,” “computer vision,” “robotic surgery,” and “real-world implementation.” Reference lists of key articles were manually screened to identify additional relevant studies.

We included original research, prospective and retrospective cohort studies, implementation studies, and high-quality systematic reviews reporting AI applications relevant to adult cardiac surgery or closely adjacent cardiovascular domains. Case reports, purely theoretical modeling studies without clinical data, and pediatric-only studies were excluded.

Given the heterogeneity of study designs and outcomes, a formal systematic review or meta-analysis was not performed. Instead, evidence was synthesized qualitatively, with explicit distinction between retrospective model development, prospective observational use, and deployed systems demonstrating measurable clinical or operational impact.

### Definition of Real-World Deployment and Evidence Maturity

For the purposes of this review, real-world deployment is defined as the use of an AI system within an operational clinical environment, beyond isolated algorithm development, with direct interaction with clinicians or integration into clinical workflows. To clarify the maturity and translational relevance of the evidence, AI applications discussed in this manuscript are categorized into three levels:

(1) Retrospective model-development studies, in which algorithms are trained and validated using historical datasets without real-time clinical use;

(2) Prospective observational or silent-deployment studies, where AI tools generate predictions in real time but do not actively influence clinical decision-making; and

(3) Implemented clinical systems, in which AI outputs are integrated into care pathways and associated with measurable effects on clinical outcomes, workflow efficiency, or resource utilization.

Throughout the manuscript, this framework is used to distinguish proof-of-concept modeling from clinically deployed systems, allowing readers to assess the strength and maturity of evidence supporting each AI application.

## 3. Artificial Neural Networks (ANNs) as the Basis for AI Models

### 3.1. Conceptual Foundations of Clinical AI

**Figure 1:**
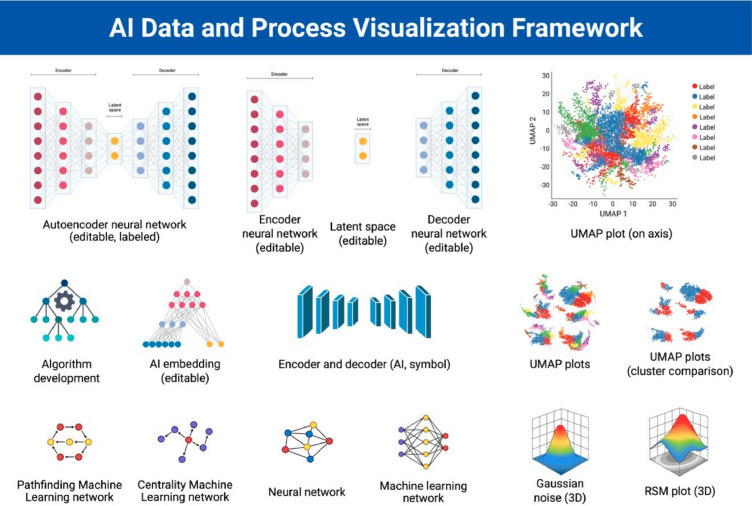
AI Data and Process Visualization Framework.

Understanding the clinical applications of AI in adult cardiac surgery requires a basic appreciation of how modern AI systems function. Figure 1 demonstrates the framework for AI data and processes. At the core of contemporary AI are artificial neural networks, computational structures inspired by the architecture of biological neurons. These networks are composed of layers of interconnected nodes that transmit information through weighted connections, analogous to synapses in the brain. As the network processes data, it iteratively adjusts these weights, thus strengthening some connections and diminishing others, to improve the accuracy of its predictions. This adaptive process underlies what is commonly described as “learning” in AI systems [10].

Central to this learning mechanism are loss functions and backpropagation. The loss function quantifies how far the model’s predicted output deviates from the ground-truth answer. Backpropagation then systematically adjusts the weights throughout the network to minimize this loss, allowing the model to gradually improve its performance on future predictions [11,12]. Through these cycles, neural networks become capable of recognizing patterns in large and complex datasets, whether those patterns involve imaging features, physiologic trends, or combinations of multimodal patient information.

AI training approaches are typically divided into supervised, unsupervised, and reinforcement learning paradigms. Supervised learning relies on labeled datasets in which the correct outputs are known in advance. The model learns to associate specific inputs with desired outputs, making this approach particularly useful for tasks such as predicting postoperative complications, classifying imaging abnormalities, or generating individualized risk scores [13].

Unsupervised learning operates without labels. Instead, the model identifies hidden patterns, clusters, or relationships embedded within the data. This approach is especially powerful in cardiology, where unsupervised models have uncovered novel disease phenotypes, subtle ECG signatures, and prognostic subgroups not captured by traditional risk scores [14]. In contrast, reinforcement learning enables an AI agent to learn optimal strategies through trial and error, receiving rewards for desirable actions and penalties for suboptimal ones. Although still in early development in cardiovascular medicine, reinforcement learning has significant potential for dynamic decision-making environments, such as optimizing real-time hemodynamic management or tailoring postoperative care pathways [15].

A key limitation of earlier AI systems was unimodality, defined as the ability to process only one type of input at a time. Cardiac surgery, by contrast, relies on the integration of imaging, physiology, surgical videos, laboratory values, and text-based clinical documentation. Recent advances in multimodal AI have allowed models to incorporate and synthesize information across these diverse data streams, generating richer, context-aware outputs. Such multimodal models are particularly promising for cardiac surgical applications, where decisions often hinge on the convergence of structural imaging, functional assessments, and intraoperative findings [16].

**Figure 2:**
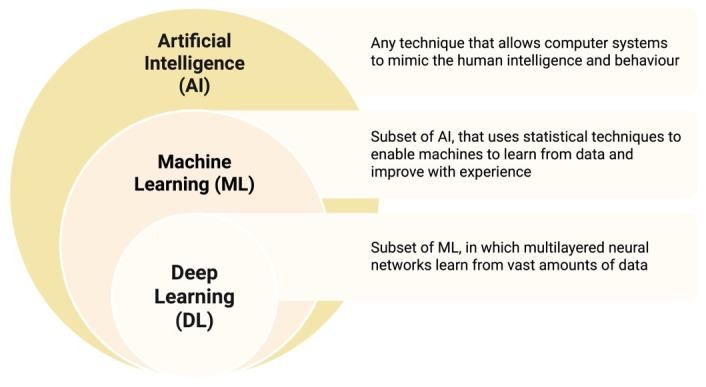
Hierarchical Relationship Between Artificial Intelligence, Machine Learning, and Deep Learning.

Together, these foundational concepts illustrate how modern AI systems learn, adapt, and integrate information through the different stages of deep learning (DL), machine learning (ML), and finally AI (Figure 2). They form the technical basis upon which contemporary cardiac surgical AI tools have been built, ranging from risk prediction algorithms to virtual simulators and intraoperative decision-support systems. This grounding is essential for understanding the clinical advances described in subsequent sections of this review.

### 3.2. Core AI Architectures Used in Cardiovascular Medicine and Surgery

While artificial neural networks form the conceptual foundation of modern AI, several specialized architectures have emerged as particularly relevant to cardiovascular medicine and cardiac surgery. Each architecture is suited to distinct data modalities and clinical tasks.

Convolutional neural networks (CNNs) are optimized for spatial data and are the dominant architecture for medical imaging and waveform interpretation. In cardiovascular medicine, CNNs underpin AI-enabled ECG analysis, echocardiographic video interpretation, and automated CT and MRI segmentation [10]. By applying convolutional filters across spatially or temporally structured inputs, CNNs can detect subtle patterns, such as latent ventricular dysfunction on ECGs or regional wall-motion abnormalities on echocardiography, that may be imperceptible to human observers [14]. These properties explain their widespread use in preoperative diagnostics, structural heart planning, and aortic imaging applications discussed in this review.

Transformer-based architectures, originally developed for natural language processing, have gained increasing prominence in healthcare due to their ability to model long-range dependencies and complex temporal relationships. Unlike CNNs, transformers use attention mechanisms to dynamically weight the importance of different input features over time [16]. This makes them particularly well suited for longitudinal electronic health record data, high-frequency physiologic waveforms, and multimodal integration of labs, vitals, imaging, and clinical notes. In perioperative and intensive care settings, transformer-based models have demonstrated strong performance in early-warning systems, postoperative complication prediction, and real-time deterioration forecasting.

Ensemble and tree-based methods, such as random forests and gradient-boosted decision trees (e.g., XGBoost), remain among the most commonly deployed AI models in cardiac surgery due to their robustness, interpretability, and strong performance on structured clinical data [14,16]. These models combine multiple weak learners to capture nonlinear interactions between variables while reducing overfitting. Ensemble approaches have been widely applied to mortality and morbidity prediction, acute kidney injury forecasting, and cost-outlier identification, often outperforming traditional regression-based risk models while offering greater transparency than deep neural networks.

Importantly, many contemporary AI systems in cardiac surgery employ hybrid or multimodal architectures, combining CNNs for imaging, transformers for time-series and longitudinal data, and ensemble models for structured variables. This architectural diversity reflects the inherently multimodal nature of cardiac surgical care and enables AI systems to generate more comprehensive, context-aware predictions across the perioperative continuum.

## 4. AI in Preoperative Diagnostics and Risk Stratification

The examples discussed below span the full spectrum of AI evidence maturity, from retrospective model-development studies to prospectively evaluated and clinically implemented systems, as defined in Section 2.

### 4.1. Enhanced Imaging Interpretation

AI has significantly advanced the interpretation of cardiovascular imaging and electrophysiologic data by detecting latent disease signatures that are often invisible to human observers. Deep learning applied to routine diagnostics, such as the standard 12-lead electrocardiogram (ECG), can uncover subclinical structural and functional abnormalities. One of the most notable examples comes from Mayo Clinic, where a convolutional neural network trained to identify left ventricular (LV) dysfunction (EF < 35%) demonstrated approximately 85% accuracy, even in individuals without overt cardiomyopathy [17]. Importantly, patients flagged as high-risk by this “AI-ECG” had a nearly four-fold higher likelihood of developing future LV dysfunction [17].

Building on this work, Attia and colleagues introduced the concept of an “AI-ECG age,” an algorithmic measure of biological cardiac aging. In a cohort of 13,808 patients undergoing CABG, those with an “older” AI-ECG age experienced higher operative complications and markedly worse long-term survival [18]. Five-year survival was 86.2% in patients with a normal AI-ECG vs. 71.4% in those with an abnormal AI-ECG; ten-year survival was 68.2% vs. 45.1%, respectively [18]. These results highlight the role of AI-enabled electrophysiologic biomarkers in refining preoperative cardiac risk assessment beyond conventional scoring systems.

AI has also expanded capabilities in echocardiography and cardiac MRI. At Stanford, Ouyang et al. developed a video-based deep learning model capable of analyzing dynamic echocardiographic clips to predict right ventricular failure after left ventricular assist device (LVAD) implantation [19]. In a 941-patient multicenter study, the AI model achieved an area under the curve (AUC) score, a performance metric used to assess effectiveness, of 0.73, surpassing both standard clinical risk scores (AUC ~0.60) and expert cardiologist assessment (AUC ~0.55) [19]. Early identification of high RV-failure risk allows surgeons to modify operative strategy, consider temporary RV support, or optimize timing of LVAD implantation.

Similarly, deep neural networks have demonstrated expert-level accuracy in distinguishing cardiac amyloidosis vs. hypertrophic cardiomyopathy on cardiac MRI [20], while AI-ECG models can detect previously unrecognized hypertrophic cardiomyopathy in asymptomatic individuals [21]. These diagnostic advances directly impact preoperative planning by influencing decisions regarding valve surgery, myectomy, transplant evaluation, or medical optimization. In parallel, natural language processing (NLP) systems are increasingly used to extract meaningful clinical variables from large volumes of unstructured imaging and electronic health record (EHR) data. Rumshisky et al. demonstrated that AI-based early-warning systems, trained using routine EHR inputs, can predict physiologic deterioration earlier than traditional alerts [5].

### 4.2. Personalized Risk Prediction

Traditional regression-based tools such as EuroSCORE II and the STS risk models remain foundational in surgical practice but are limited by linear assumptions and fixed variable interactions [1,2]. Machine learning methods, by contrast, integrate high-dimensional clinical, imaging, and physiologic data, enabling the discovery of nonlinear relationships and previously unrecognized risk patterns [3]. In fact, artificial neural networks were among the earliest ML tools applied in cardiac surgery. Nilsson et al. used an ANN to automatically select the 34 most predictive variables (from 72 candidates) for operative mortality, significantly outperforming EuroSCORE II [22]. In LVAD surgery, Kilic and colleagues compared logistic regression vs. ML survival models and demonstrated superior accuracy and calibration of ML predictors for 90-day and 1-year mortality [23].

In heart transplantation, Yoon et al. introduced a “tree-of-predictors” model that offered more precise, individualized survival prediction compared with traditional approaches [24]. Such models identify high-risk phenotypes that are not captured by linear risk scores, enabling better candidate selection and tailored perioperative strategies.

More recently, an XGBoost model trained on 4874 cardiac surgery patients from Mayo Clinic and Baylor outperformed STS calculators in predicting mortality, major morbidity, and high-cost outlier cases [25]. This model captured complex nonlinear interactions, including lab trajectories, physiologic instability, and subtle imaging patterns, associated with complications such as acute kidney injury (AKI), prolonged ventilation, sepsis, and postoperative delirium.

Complementing these predictive tools, machine learning–based early-warning systems have demonstrated measurable real-world impact. Shimabukuro et al. showed that an ML-driven severe sepsis prediction tool reduced ICU transfer delays and mortality [7]. Similar early-warning frameworks applied preoperatively can guide optimization, refine operative timing, and inform postoperative ICU resource allocation. Furthermore, as documented by Johnson and colleagues, many modern AI risk tools draw from existing EHR data or even a single ECG, enabling seamless integration into clinical workflows without increasing documentation burden [19]. Surgeons have reported using such AI-derived risk estimates during preoperative consultations to provide personalized complication profiles and support shared decision-making [25]. These tools, once validated, can complement existing calculators and help tailor surgical planning to each patient’s risk signature.

### 4.3. Surgical Planning and Virtual Simulation

AI-driven imaging reconstruction, augmented reality (AR), and virtual reality (VR) platforms are increasingly shaping preoperative planning in adult cardiac surgery by providing surgeons with deeper anatomic insight before entering the operating room. Advanced image-processing algorithms enable rapid, high-fidelity segmentation of cardiac structures from CT or MRI scans, producing patient-specific three-dimensional (3D) models for tactile review, procedural rehearsal, and operative strategy refinement. Machine learning and computer vision markedly improve the speed and accuracy of these reconstructions, allowing surgeons to interact with realistic simulations of complex anatomy [26,27,28].

Integration of AI-based segmentation with 3D printing has been particularly useful in complex cardiac and structural heart cases. Investigators at the University of Minnesota demonstrated that combining high-resolution imaging, computer vision, and 3D printing yields life-like cardiac models with precise replication of valvular, coronary, and chamber anatomy, enabling rehearsal of challenging operations and identification of unforeseen hazards [26]. Similar work in congenital and structural heart disease shows that hands-on preoperative manipulation of models improves surgeon confidence, enhances communication with the surgical team, and may reduce operative uncertainty [27,28].

Virtual reality systems further expand this precision by generating immersive, interactive environments in which surgeons can “enter” the patient’s anatomy. In a reported series using PulmoVR, a VR-based segmentectomy planning system, surgeons altered their operative plan in 4 of 10 cases after reviewing the 3D virtual reconstruction [29]. Analogous VR, AR, and mixed-reality approaches are increasingly being applied to cardiac tumors, anomalous coronary pathways, aortic root variants, and complex redo-valve cases where standard imaging may not adequately reveal adhesions or anatomic hazards [30,31,32].

Mixed-reality technologies, particularly holographic overlays, are entering the cardiac operating room as well. The Cleveland Clinic Heart & Vascular Institute piloted the use of Microsoft HoloLens projections derived from preoperative computed tomography (CT) scans to assist intraoperative navigation [33]. Surgeons viewed real-time holographic reconstructions of patient-specific cardiac structures overlaid onto the operative field, enhancing orientation during reoperative sternotomy, coronary reimplantation, and minimally invasive valve procedures. Early experience suggests that these tools may help anticipate anatomic pitfalls, shorten cognitive interpretation time, therefore reducing intraoperative variability [33,34].

The common thread linking these innovations is the role of AI in image segmentation, reconstruction, and simulation. Traditional manual segmentation can require hours of expert time, whereas AI-driven workflows can produce accurate cardiac 3D models within minutes, enabling same-day surgical planning [28,35]. These technologies are beginning to converge with intraoperative navigation systems, raising the possibility of real-time AI-assisted annotation of surgical anatomy to enhance operative precision and individualized surgical strategy (Table 1).

### 4.4. Comparative Performance of AI, Traditional Risk Models, and Clinician Assessment

A central question for real-world adoption of artificial intelligence in cardiovascular medicine is whether AI systems meaningfully outperform traditional methods or human expert assessment. Across multiple domains, growing evidence suggests that AI offers incremental value when applied to data-intensive tasks characterized by nonlinear interactions, high-dimensional inputs, or subtle pattern recognition.

In preoperative risk stratification, machine-learning and deep-learning models have consistently demonstrated superior discrimination and calibration compared with conventional regression-based tools such as EuroSCORE II and the STS risk calculators [22]. Meta-analytic data indicate that AI-based models improve prediction of mortality and major morbidity across diverse cardiac surgical populations, particularly in high-risk subgroups where traditional scores tend to underperform. Importantly, these gains arise not from replacement of clinical judgment, but from the ability of AI to integrate complex variable interactions and temporal trends that are difficult to model manually.

Comparisons between AI and clinician performance are most mature in diagnostic applications (Table 2). In electrocardiography and cardiac imaging, deep-learning models have matched or exceeded expert-level accuracy in detecting left ventricular dysfunction, hypertrophic cardiomyopathy, and infiltrative disease, while also identifying prognostically relevant signatures invisible to human interpretation. These findings, largely derived from cardiology cohorts, provide important translational insight for cardiac surgery, where preoperative diagnostics directly influence operative strategy, timing, and risk counseling. A recent review of AI deployment in cardiovascular medicine highlighted multiple real-world examples in which AI-assisted workflows improved diagnostic accuracy, efficiency, or clinical decision-making compared with conventional approaches, underscoring the broader relevance of these tools beyond isolated modeling studies [36].

In contrast, AI appears less advantageous in domains dominated by low-dimensional decision rules or well-established protocols, emphasizing the importance of targeted, task-specific deployment. Collectively, existing evidence supports a model of AI-augmented care, in which artificial intelligence complements rather than replaces clinician expertise, enhancing performance in areas where human cognition is intrinsically limited while preserving clinician oversight in high-stakes decision-making.

## 5. Intraoperative AI Assistance and Robotic Surgery

### 5.1. Robotics and Automation

Robotic surgery is now well established in adult cardiac procedures, including robotic-assisted CABG and mitral valve repair, but contemporary systems remain largely tele-operated, with surgeons controlling instruments at the console. The integration of AI into robotic platforms is expected to shift these systems from purely manual extensions of the surgeon toward smart, semi-autonomous assistants. Several technological breakthroughs illustrate this evolution.

One of the most prominent advances is the work from Johns Hopkins University, where investigators developed the Smart Tissue Autonomous Robot (STAR), a platform that uses computer vision, machine learning, and force-sensing algorithms to perform soft-tissue suturing [37]. In preclinical models, STAR autonomously executed intestinal anastomoses with greater precision and consistency than expert human surgeons, achieving superior spacing and tension uniformity of sutures [37,38]. Although fully autonomous cardiac anastomosis in humans remains distant, these foundational studies demonstrate that AI-enabled robotic systems can outperform conventional manual techniques in controlled environments.

Current-generation clinical platforms are also incorporating AI-driven features. Robotic systems now routinely include motion scaling, tremor suppression, and intelligent tool-path smoothing algorithms that enhance precision and minimize human error [39,40]. Advanced computer-vision modules are being tested for real-time identification of anatomical landmarks, such as coronary ostia, valve annuli, or atrioventricular conduction pathways, alerting the surgeon when instruments approach critical structures [41]. These guidance systems may reduce avoidable injuries, improve suture placement, and increase reproducibility during minimally invasive or robotic cardiac surgery.

AI is also transforming intraoperative perception. Enhanced vision systems using machine learning can automatically detect tissue planes, bleeding points, suture lines, and graft orientation, providing the surgeon with augmented situational awareness [42,43]. Experimental work in minimally invasive CAD (computer-assisted design) surgery shows that AI-based fluorescence analysis and perfusion mapping may soon assist with graft assessment, coronary flow evaluation, and valve inspection during live cases [44].

Another rapidly emerging category is intraoperative AI motion analysis, sometimes termed “OR Black Box” technology. These systems use overhead cameras and sensor-based tracking to capture surgical movements, instrument trajectories, and operative errors. Seminal studies by collaborators at the University of Toronto and other centers demonstrated that AI-driven analysis can identify inefficient or hazardous motions with high accuracy, correlating specific gesture patterns with technical errors or adverse events [45,46]. Feedback from such systems allows surgeons to adjust technique in real time or during postoperative debriefing, leading to measurable improvements in operative safety and consistency.

Importantly, experts emphasize that the purpose of intraoperative AI is not to replace the cardiac surgeon but to serve as a high-reliability co-pilots, by processing visual, kinematic, and physiologic data far faster than a human and surfacing key insights at the moment they matter most [39,47]. Although outcome-level evidence in cardiac surgery is still developing, early experiences show reductions in technical errors, improved standardization of complex maneuvers, and enhanced team situational awareness. As AI-enabled robotics continue to advance, from intelligent tool tracking to semi-autonomous suturing, the field is gradually progressing toward collaborative autonomy, in which surgeons maintain full control while AI augments precision, safety, and intraoperative decision-making.

### 5.2. AI-Enhanced Hemodynamic Management

One of the most tangible intraoperative benefits of AI in adult cardiac surgery has emerged in the field of hemodynamic optimization. Cardiac operations require meticulous control of perfusion and blood pressure, as even brief periods of intraoperative hypotension can contribute to myocardial injury, stroke, or AKI. Historically, anesthesiologists managed hemodynamics reactively, responding to drops in arterial pressure only after they occurred. AI has fundamentally shifted this paradigm by allowing clinicians to act before instability develops.

The best example is the Hypotension Prediction Index (HPI), a machine-learning algorithm that continuously analyzes the arterial waveform to forecast the onset of hypotension minutes before it occurs [48,49,50]. Originally validated in non-cardiac surgery, HPI has now entered the realm of cardiac Enhanced Recovery After Surgery (ERAS) protocols. A recent multi-center evaluation examined more than 600 cardiac surgery patients across three institutions and found that adding HPI-guided management produced meaningful improvements in recovery [51]. Patients managed with AI-augmented care experienced a reduction in total postoperative ventilation time of approximately 4.4 h (from 13.5 to 9.1 h, *p* = 0.03), and although the reduction in ICU stay, about 6.8 h, did not reach statistical significance, it suggested a trend toward faster stabilization [52]. The incidence of AKI remained unchanged in this early report, but the authors noted that improved perfusion stability could yield renal benefits in larger cohorts.

Clinicians who have integrated HPI into their practice often describe it as a new form of “anticipatory vital sign,” offering predictive information that complements traditional monitoring modalities [51]. By warning clinicians before a hemodynamic downturn, the algorithm allows earlier vasopressor adjustments, more controlled fluid administration, and smoother separation from cardiopulmonary bypass, changes that collectively contribute to more stable postoperative trajectories. Beyond arterial waveform prediction, research groups are beginning to explore AI-enabled perfusion systems that could automatically adjust cardiopulmonary bypass flow, cooling rates, and hematocrit targets within safe limits. Closed-loop systems already exist in critical care for anesthetic depth or glycemic control, and similar automated feedback models are now being adapted to cardiac perfusion management [53,54,55]. These developments reflect a broader trend: AI is gradually transforming intraoperative hemodynamic care from a reactive process into a predictive and potentially semi-autonomous system that supports the clinical team in maintaining optimal physiologic conditions.

### 5.3. Intraoperative Computer Vision

Another major frontier of intraoperative AI is the application of computer vision, a domain in which algorithms analyze live surgical video to extract quantitative data that go far beyond the limits of human perception. The most striking illustration of this comes from a thoracic surgery study in Beijing, where investigators trained a computer vision algorithm to identify and quantify blood pixels in the operative field during thoracoscopic lobectomies [56]. In a cohort of 275 patients, the proportion of blood pixels identified on a frame-by-frame basis strongly predicted key postoperative outcomes. Higher intraoperative bleeding signatures were associated with increased postoperative chest-tube drainage, longer drainage duration, and greater intraoperative blood loss. Even after multivariable adjustment, the computer vision-derived bleeding measure remained an independent predictor of prolonged drainage duration (odds ratio ~1.003 per unit, *p* = 0.017) [56]. This study was the first to demonstrate that intraoperative AI video analysis could meaningfully link real-time operative conditions with downstream patient outcomes.

While this early work was conducted in thoracic surgery, its implications for adult cardiac surgery are profound. Bleeding remains a central concern in procedures such as aortic operations, reoperative sternotomies, and minimally invasive valve repairs. A computer vision system capable of continuously quantifying subtle blood accumulation, either in the field or on surgical sponges, could alert the operative team to early signs of excessive blood loss, enabling timely correction before instability manifests. Moreover, because cardiac surgery often involves delicate dissection planes, computer vision-based tracking of instrument movements and tissue planes could identify when a trajectory is veering too close to a coronary artery, the conduction system, or a thin-walled cardiac chamber.

These capabilities align closely with the emerging discipline of surgical data science, which integrates computer vision, motion analysis, and multimodal intraoperative data to improve situational awareness and reduce intraoperative errors [43,45,57]. Early “OR Black Box” technologies already demonstrate that real-time AI analysis of surgical video and instrument motion can detect errors, deviations from ideal technique, and potentially unsafe patterns during laparoscopic or robotic procedures [45,46]. Translating these systems into cardiac surgery, where the margin for error is even smaller, could enhance safety by providing continuous, objective surveillance of the operative field.

Researchers have also begun adapting computer vision algorithms specifically for cardiovascular procedures, including tools that automatically identify coronary landmarks during robotic CABG, quantify myocardial perfusion using fluorescence imaging, or detect left ventricular distension during minimally invasive surgery [58,59,60]. These early efforts suggest that computer vision will likely evolve into a continuous, automated “set of eyes” in the operating room, providing the surgical team with real-time alerts, quality assurance, and actionable insights that augment their technical performance. As these systems mature, computer vision has the potential to redefine intraoperative decision-making by turning visual data, previously interpreted solely by humans, into quantitative, predictive information that enhances surgical safety and precision.

### 5.4. AI in Structural Heart Surgery

AI has played an increasingly influential role in the planning, execution, and optimization of structural heart interventions, where procedural success relies on a precise understanding of complex three-dimensional anatomy and dynamic physiology. Structural heart surgery, particularly transcatheter aortic valve replacement (TAVR), transcatheter mitral procedures including transcatheter mitral valve replacement (TMVR) and transcatheter edge-to-edge repair (TEER), and left atrial appendage occlusion (LAAO), requires accurate preoperative imaging, computational modeling, and device selection. AI has enabled major advances in each of these domains.

One of the most mature applications is AI-assisted CT analysis for TAVR planning, where deep learning algorithms automate annular sizing, leaflet calcification quantification, coronary ostial height assessment, and prediction of paravalvular leak (PVL). Traditional TAVR planning involves time-intensive manual segmentation; AI-based tools now perform these tasks within seconds and with expert-level reproducibility [61,62]. In multicenter evaluations, ML-driven annular measurement showed excellent agreement with expert readers and reduced interobserver variability, streamlining procedural workflows [62].

Beyond anatomic characterization, machine-learning models have been developed to predict TAVR complications, including conduction disturbances requiring permanent pacemaker implantation, valve embolization, and moderate-to-severe PVL. By integrating CT morphology, clinical variables, and valve selection parameters, these models outperform conventional logistic models and offer individualized risk prediction [63]. Early work has even shown that AI can recommend optimal valve size and type (balloon-expandable vs. self-expanding) based on patient-specific anatomy, achieving concordance levels comparable to multidisciplinary heart-team decisions [64].

In the mitral valve domain, AI has transformed TMVR planning, particularly in the prediction of left ventricular outflow tract (LVOT) obstruction—one of the most feared complications. Deep learning tools automatically identify anatomic markers associated with obstruction risk and simulate “neo-LVOT” geometry after virtual valve deployment [65]. Virtual implantation platforms allow teams to test multiple valve positions and device types preoperatively, making them invaluable in redo mitral cases, heavy annular calcification, and complex subvalvular anatomy where conventional CT assessment may underestimate obstruction risk [65,66].

AI-based segmentation and motion-tracking also enhance transcatheter tricuspid valve intervention planning, where anatomic heterogeneity and limited leaflet visualization have historically hindered reproducibility. ML algorithms provide automated assessment of annular shape, leaflet tethering, right-ventricular strain, and coaptation gaps, improving device selection and trajectory planning [67]. As dedicated tricuspid devices expand in use, AI-guided planning is likely to become standard.

Left atrial appendage occlusion has similarly benefited from AI tools that generate virtual LAAO simulations, offering device sizing recommendations and predicting landing-zone seal quality. Several groups have shown that AI-guided LAAO planning reduces device-related leaks and procedure time by producing more accurate preoperative sizing and implantation angles [68].

Finally, the integration of AI with intraoperative computer-vision platforms is beginning to influence structural interventions. Vision-based systems capable of recognizing catheters, guidewires, annular planes, leaflet grasping zones, and deployed valves are under development [69]. These systems are expected to provide real-time, image-based feedback during transseptal puncture, annular positioning, leaflet capture in TEER procedures, or valve deployment, acting as a procedural “co-pilot.”

Together, these innovations illustrate how AI is reshaping structural heart surgery by enhancing preoperative accuracy, improving device selection, and supporting real-time decision-making during complex transcatheter interventions. As structural heart programs continue to expand, AI will play an indispensable role in improving safety, reproducibility, and individualized procedural planning.

### 5.5. AI in Aortic Surgery

AI is increasingly being applied to aortic surgery, a field where outcomes depend on precise risk stratification, accurate measurement of aortic geometry, and prediction of catastrophic complications such as dissection and rupture. Traditional metrics such as maximal aortic diameter often fail to capture the complex biomechanical forces that determine wall instability. Machine-learning models have therefore been developed to integrate geometric parameters, wall stress distribution, genetic variants, biomarkers, and imaging-derived phenotypes to improve prediction of adverse events [70].

Several groups have demonstrated the value of AI-assisted aortic segmentation and biomechanical modeling using CT and magnetic resonance imaging (MRI). Deep learning tools can automatically measure aortic length, tortuosity, curvature, cross-sectional area, and regional wall thickness, features that correlate with dissection risk but are difficult to capture manually [71,72]. These AI-derived shape descriptors have outperformed diameter alone in predicting acute type A aortic dissection (ATAAD) and rapid aneurysm growth [71].

In patients undergoing open or endovascular aortic repair, AI is transforming operative planning by enabling virtual simulation of graft sizing, optimal clamp placement, and pathways for cannulation. For thoracic endovascular aortic repair (TEVAR), ML-driven analysis can predict endoleak risk, graft migration, and landing zone adequacy with higher accuracy than traditional rule-based criteria [73]. Similarly, AI-based hemodynamic simulations, using computational flow dynamics integrated with machine learning, allow surgeons to anticipate areas of high wall shear stress or flow stagnation, predictors of postoperative complications and reintervention [74].

AI is also emerging as a valuable tool in postoperative risk prediction. Models trained on large aortic surgery datasets have demonstrated improved accuracy in forecasting acute kidney injury, prolonged ventilation, stroke, and long-term mortality compared with EuroSCORE II or standard STS risk tools [75]. Early efforts using natural language processing (NLP) have shown promise in extracting aortic pathology descriptors from operative notes and radiology reports to automatically populate surveillance registries [76].

As more centers collect granular perioperative and imaging data, AI-driven insights are expected to refine patient selection, optimize timing of intervention, and support precision aortic surgery by leveraging multimodal data—including biomarkers, phenotypes, and molecular signatures associated with aneurysm degeneration.

### 5.6. Digital and Hybrid Operating Room Ecosystems

The emergence of AI-enabled digital operating rooms (D-ORs) and hybrid OR ecosystems is transforming the surgical environment into an interconnected, data-rich space. These ORs integrate imaging systems, robotic platforms, anesthesia monitors, perfusion consoles, and environmental sensors into a unified digital infrastructure capable of real-time data exchange and AI analysis [77]. Modern hybrid ORs, equipped with cone-beam CT, advanced fluoroscopy, 3D rotational angiography, and robotic C-arm systems, generate large streams of intraoperative imaging data. AI-driven reconstruction can then produce real-time 3D maps of the cardiac and aortic anatomy, guiding graft placement, valve deployment, or endovascular stent positioning without interrupting the workflow [78]. Computer-vision systems integrated with ceiling-mounted cameras can continuously monitor the OR environment, tracking instrument locations, handoffs, sterility boundaries, and team movement patterns. Early investigations show that AI can detect workflow deviations associated with prolonged bypass time, miscommunication events, or increased risk of intraoperative error [79]. In the anesthesia domain, smart ORs integrate physiologic monitoring with AI prediction models to guide vasopressor titration, depth-of-anesthesia control, and extubation readiness. Perfusion consoles are increasingly equipped with intelligent decision-support systems that evaluate hemodynamic trends, venous saturation, blood-gas data, and pump flow performance to support perfusionists during complex cardiopulmonary bypass (CPB) runs [80].

The ultimate vision is a fully integrated AI-orchestrated hybrid OR, in which imaging, robotics, perfusion, and anesthesia systems share data seamlessly. Such systems could provide moment-to-moment guidance—alerting teams about tissue tension during cannulation, showing holographic overlays during complex dissections, or predicting transitions toward hemodynamic instability. This ecosystem would support enhanced situational awareness, reduce human-factor error, and enable a level of procedural precision beyond human-only capabilities.

### 5.7. Cost-Effectiveness of AI Adoption in Cardiac Surgery

As AI systems transition from experimental tools to clinical deployment, their cost-effectiveness has become a central consideration for health systems [81]. Importantly, the current economic evidence base in cardiac surgery includes a limited number of empirically measured cost analyses, complemented by a broader body of modeled or extrapolated data from cardiology, critical care, and other surgical specialties [82]. Distinguishing between these evidence types is essential to accurately assess the present and future economic value of AI-enabled cardiac surgical care.

### 5.8. Empirically Demonstrated Economic Impact in Cardiac Surgery

Direct cost analyses specific to cardiac surgery remain limited but are beginning to emerge. AI-augmented perioperative strategies that reduce postoperative ventilation duration, ICU length of stay, or major complications offer the clearest pathways to measurable cost savings. For example, implementation of Hypotension Prediction Index–guided hemodynamic management within cardiac ERAS pathways has been associated with shorter ventilation times and trends toward reduced ICU utilization, outcomes that are consistently linked to lower hospitalization costs [83].

Similarly, machine-learning–based early-warning systems that reduce failure-to-rescue events or unplanned ICU transfers may generate downstream economic benefit by preventing high-cost complications, although formal cost-effectiveness analyses within cardiac surgery populations remain sparse. Where available, reported savings are largely indirect, inferred from reductions in length of stay, complication burden, or resource utilization rather than from prospective cost-accounting studies.

AI can also generate savings by improving resource allocation. For example:

- OR scheduling algorithms can decrease turnover time and increase case throughput [84]
- Predictive modeling for ICU bed demand helps prevent costly delays or cancellations [85]
- Automated imaging and reporting reduce radiologist and surgeon workload, freeing personnel for higher-value tasks [86]

Although randomized trials evaluating cost-effectiveness in cardiac surgery remain limited, early economic analyses in cardiology and surgical specialties consistently show net cost savings when AI reduces complication burden, procedural variability, and workflow inefficiencies.

As adoption scales (Table 3), AI’s cost-effectiveness is expected to improve further due to economies of scale, cloud-based deployment, and integration into existing EHR systems, thus positioning AI not only as a clinical adjunct but also as a financially sustainable strategy for high-volume cardiac surgery programs.

## 6. Postoperative AI Monitoring and ICU Decision Support

Postoperative care in adult cardiac surgery is a data-dense and clinically volatile phase in which early deterioration can occur rapidly and unpredictably. Small physiologic deviations—subtle changes in heart rate variability, microcirculatory perfusion, ventilatory parameters, or inflammatory markers—frequently precede overt clinical decline by hours. Traditional monitoring systems rely on threshold-based alarms and intermittent clinician assessment, which may delay recognition of complications such as AKI, low-cardiac-output syndrome (LCOS), postoperative hemorrhage, or sepsis. Artificial intelligence (AI), particularly machine learning and deep learning models, has begun to address these limitations by enabling continuous, predictive analysis of postoperative physiology and electronic health record (EHR) data.

### 6.1. Predicting Postoperative Complications

AI models have demonstrated strong performance in predicting major postoperative complications earlier than standard monitoring tools. Several groups have trained supervised-learning algorithms to forecast AKI following cardiac surgery using preoperative labs, intraoperative hemodynamics, and early postoperative trends. Gradient-boosting and recurrent neural network models have consistently outperformed traditional logistic regression and clinical prediction tools in forecasting AKI within the first 48 h after surgery [87,88]. These models can incorporate thousands of data points, including time-series blood pressure variability, cardiopulmonary bypass parameters, and urine output dynamics, providing clinicians with actionable, real-time probability scores. Similar approaches have been developed to predict LCOS, prolonged ventilation, and new-onset atrial fibrillation. Deep learning networks analyzing minute-by-minute physiologic waveforms have shown strong early-warning accuracy for impending LCOS or need for mechanical circulatory support [89]. Machine-learning predictors for postoperative AF, integrating atrial strain, autonomic signatures, and biomarker profiles, have reduced AF incidence in prospective pilot studies by guiding prophylactic therapy in high-risk patients [90].

### 6.2. Early Warning Systems and Real-Time Deterioration Predictions

AI-based early-warning systems (EWS) have been among the most impactful postoperative innovations to date. Unlike rule-based systems (e.g., MEWS), ML-driven tools continuously update risk estimates using streaming vital signs, laboratory data, telemetry, and ventilator parameters.

The Sepsis Prediction Model (SPM) and Epic’s Deterioration Index, though not cardiac-specific, have been validated in cardiothoracic populations. They identify sepsis and hemodynamic collapse hours earlier than bedside evaluations, reducing progression to septic shock [91]. Similarly, the eCART algorithm, using vital signs, nursing assessments, and labs, has demonstrated significant improvement in predicting unplanned ICU transfers and cardiac arrest [92].

In cardiac surgery ICUs, real-time waveform analytics combined with ML can identify impending respiratory failure, prompting earlier extubation planning or avoidance of premature extubation [93]. Closed-loop ventilator systems using reinforcement learning have been tested experimentally and may soon offer automated weaning protocols based on predicted physiologic trajectories [94].

### 6.3. AI in ICU Hemodynamic and Perfusion Management

Postoperative hemodynamic instability remains a leading driver of morbidity. AI algorithms trained on arterial waveform morphology, cardiac output trends, and vasoactive medication profiles can forecast hypotension, vasoplegia, or volume responsiveness earlier than traditional metrics.

Building on intraoperative evidence, postoperative deployment of the Hypotension Prediction Index (HPI) has shown feasibility in pilot cardiac ICU settings, allowing clinicians to intervene before blood pressure declines [95]. Reinforcement-learning perfusion models trained on CPB data are also being adapted for postoperative care, identifying optimal MAP targets individualized to each patient’s physiology [96]. AI-driven microvascular monitoring, using tissue oximetry and perfusion imaging combined with ML, has demonstrated promise in detecting early organ malperfusion even when macrocirculatory parameters appear stable [97].

### 6.4. Computer Vision for Postoperative Surveillance

Computer-vision (CV) systems are emerging as tools for postoperative safety monitoring. Ceiling-mounted cameras integrated with CV algorithms can detect:

- Patient agitation or delirium
- Line dislodgement risk
- Falls, unassisted bed exits
- Chest-tube output saturation patterns
- Early signs of tamponade such as increased respiratory effort or body-position changes

Studies in surgical ICUs show that CV-enabled delirium detection can outperform manual nursing assessments, identifying motor agitation and facial microexpressions associated with hyperactive delirium [98]. Although early in cardiac surgery, these systems could support earlier mobilization, reduce nighttime complications, and extend nursing situational awareness.

### 6.5. AI for Postoperative Workflow Optimization and Discharge Planning

Machine-learning tools have been deployed to streamline postoperative workflow, predict discharge readiness, and reduce length of stay. Models using perioperative variables, day-to-day lab dynamics, chest-tube output trajectories, and mobility data can predict the likelihood of discharge within 24–48 h with high discrimination [99].

Predictive tools have also been developed for readmission risk, integrating social determinants of health, symptom burden, arrhythmia telemetry, and laboratory signatures. These tools have demonstrated improved accuracy over the LACE index and can guide targeted follow-up strategies [100].

Remote-monitoring systems equipped with AI (for example, arrhythmia detection, heart-rate variability analysis, or digital biomarkers of fluid status) can continuously evaluate patients after discharge. Early studies indicate that AI-enabled remote monitoring reduces unplanned readmissions and improves detection of postoperative complications such as pleural effusion, arrhythmia recurrence, or wound infection [101].

## 7. Ethical, Regulatory, and Data-Governance Challenges in Cardiac AI

As artificial intelligence becomes increasingly embedded in cardiac surgical workflows, from preoperative diagnostics to postoperative ICU monitoring, its safe and responsible integration depends on resolving a series of ethical, regulatory, and data-governance challenges. Unlike traditional medical devices, AI algorithms evolve through continuous data ingestion, retraining, and model updating, raising important concerns about transparency, accountability, and patient autonomy. Addressing these issues is essential for ensuring that AI serves as a trustworthy adjunct to cardiac clinicians while avoiding unintended harm.

### 7.1. Transparency, Explainability, and Clinical Trust

Most high-performing AI models, particularly deep neural networks, operate as “black boxes” whose internal logic is opaque even to developers. This lack of explainability poses a significant barrier in cardiac surgery, where high-stakes decisions, such as proceeding with high-risk coronary bypass, aortic root replacement, or valve intervention, require clear justification. Explainable AI (XAI) tools, including saliency maps, feature-importance rankings, and counterfactual explanations, are being developed to provide interpretable insights from complex models [102].

Studies show that clinicians are more likely to adopt AI recommendations when the model offers interpretable rationales rather than outputting isolated risk scores [103]. Improving model transparency is therefore essential for fostering surgeon trust, promoting shared decision-making, and mitigating the risk of blind overreliance on algorithmic predictions.

### 7.2. Bias, Fairness, and Equity in Model Performance

AI algorithms inherit biases present in the datasets used to train them. For cardiac surgery, where outcomes differ across race, sex, socioeconomic status, and comorbidity burden, biased models risk perpetuating or worsening disparities. Analyses of cardiovascular AI tools have demonstrated inconsistent performance among minority groups, older patients, and women due to underrepresentation in training datasets [104]. For example, predictive models for postoperative complications may underestimate risk in populations rarely represented in large registries, leading to inappropriate triage or misguided perioperative strategies. Ensuring fairness requires implementing bias-detection frameworks, stratified performance reporting, and deliberate oversampling or reweighting techniques to achieve demographic balance [105]. International guidelines now recommend fairness audits as a prerequisite for deployment of clinical AI [106].

### 7.3. Data Privacy, Security, and Consent

Cardiac AI systems rely heavily on multimodal data, such as high-resolution imaging, continuous monitoring waveforms, operative video, and EHR-derived variables. These datasets carry sensitive patient information that must be protected against unauthorized access. The use of cloud-based AI services and data-sharing across institutions raises concerns regarding compliance with Health Insurance Portability and Accountability Act (HIPAA), General Data Protection Regulation (GDPR), and local privacy laws.

Advanced encryption, differential privacy, and federated learning strategies allow model training across multiple centers while minimizing the transfer of identifiable patient data [107]. However, current informed-consent frameworks often fail to capture the dynamic and ongoing nature of AI model improvement. Emerging proposals advocate for dynamic consent models, enabling patients to control how their data are used, updated, and shared across evolving AI applications [108].

### 7.4. Regulatory Pathways for AI in Cardiac Surgery

Regulatory agencies face the challenge of overseeing AI systems that do not behave like traditional, fixed medical devices. The FDA and European Medicines Agency (EMA) have adopted new frameworks for software as a medical device (SaMD), including mechanisms for monitoring adaptive algorithms that update after deployment. The FDA’s proposed Predetermined Change Control Plan provides a pathway for manufacturers to predefine the types of algorithmic updates allowed without requiring a full recertification [109]. However, validation requirements for high-risk surgical applications remain stringent. AI tools involved in diagnosis, risk prediction, or intraoperative guidance may require multicenter trials, prospective evaluation, and continuous postmarket surveillance. Regulatory experts emphasize that safety thresholds for cardiac surgery must be exceptionally high, given the narrow margin for error and potential for catastrophic outcomes [110].

### 7.5. Liability and Accountability

The integration of AI into surgical workflows raises fundamental questions about liability. If an AI recommendation contributes to postoperative harm, such as misclassification of risk, misidentification of anatomy, or incorrect hemodynamic guidance, who is responsible: the surgeon, the institution, the AI developer, or the device manufacturer? Current legal structures generally place responsibility on the clinician as the final decision-maker, but the increasing autonomy and influence of AI systems complicate this assumption [111]. In this context, scholars have proposed shared-liability models and clearer documentation of how AI outputs influence clinical decisions. Some centers are exploring audit trails that track model versions, training datasets, and decision-support outputs to enable retrospective review [112].

### 7.6. Data Drift, Model Decay, and Revalidation

AI models degrade over time due to data drift, where patient characteristics, treatment practices, or institutional workflows evolve beyond the conditions under which the model was trained. In cardiac surgery, where device technology, anesthetic strategies, perioperative protocols, and population demographics continually evolve, model decay can rapidly erode predictive performance [113]. Continuous monitoring, periodic retraining, and rigorous revalidation processes are necessary to ensure safe, sustained performance. Many experts advocate for lifecycle-based oversight, in which models undergo quality assurance checks analogous to maintenance of surgical equipment [114].

## 8. Limitations of Current Evidence

### 8.1. Methodological, Data, and Implementation Limitations of Cardiac Surgical AI

Despite rapid advancements in artificial intelligence across adult cardiac surgery, the present evidence base remains constrained by several important methodological and practical limitations. Most published studies originate from retrospective, single-center cohorts, limiting both generalizability and external validity. Because datasets vary widely in patient demographics, comorbidity burden, imaging protocols, surgical techniques, and perioperative workflows, AI models trained in one institution frequently perform less reliably when deployed elsewhere. This issue of limited heterogeneity is well recognized in AI research more broadly, where the challenge of ensuring robust generalization across diverse settings has been repeatedly emphasized [115,116].

In cardiac surgery, center-specific practice patterns introduce concrete sources of data heterogeneity that directly affect AI model performance and external validity. For example, perfusion strategies vary widely across institutions with respect to mean arterial pressure targets, hematocrit thresholds, temperature management, and use of pulsatile versus nonpulsatile flow during cardiopulmonary bypass [53]. Models trained to predict outcomes such as acute kidney injury or low-cardiac-output syndrome may therefore internalize institution-specific perfusion signatures rather than generalizable physiologic risk factors, leading to degraded performance when deployed elsewhere.

Electronic health record (EHR) structure and data granularity represent another major barrier. Differences in waveform sampling frequency, variable naming conventions, timing of laboratory measurements, and documentation practices can substantially alter feature availability and temporal resolution. For instance, arterial waveform–based models trained on high-frequency data may fail when applied to centers that store lower-resolution signals or intermittently captured values [115]. Similarly, natural language processing models trained on operative notes from one institution often perform poorly when applied to different documentation styles or templating systems.

Outcome definition variability further complicates model generalizability. Key postoperative endpoints such as vasoplegia, delirium, low-cardiac-output syndrome, or prolonged ventilation lack universally accepted definitions in cardiac surgery. As a result, AI models may learn surrogate or proxy signals that reflect local coding or clinical culture rather than true pathophysiology, limiting their reliability across centers.

Compounding this limitation is the relative scarcity of prospective evaluation. Only a handful of AI systems in perioperative medicine have undergone prospective assessment, and virtually none have been subjected to randomized controlled trials within cardiac surgery. Even sophisticated tools that have demonstrated strong retrospective performance (for example, models predicting acute kidney injury, low-cardiac-output syndrome, or postoperative sepsis) have not yet been convincingly shown to improve outcomes when implemented in real-world practice [117]. As a result, the true clinical impact of AI-driven decision support remains largely theoretical, and the extent to which these systems alter postoperative trajectories, reduce complications, or shorten hospitalization remains uncertain.

Variability in data quality further constrains the reliability of current models. Cardiac surgery involves complex perioperative data streams, ranging from perfusion strategies and anesthetic choices to imaging acquisition and postoperative monitoring, and these elements differ significantly between centers. Without standardized definitions for key outcomes such as vasoplegia, postoperative delirium, or low-cardiac-output syndrome, AI systems may internalize center-specific patterns rather than generalizable physiologic signatures [118]. The inconsistent frequency of data acquisition, missing time-series information, and variation in EHR architectures add additional layers of complexity, often reducing the fidelity of waveform- and trend-based predictions.

Transparency in model development and reporting also remains insufficient. Many studies lack adequate detail regarding dataset composition, model architecture, hyperparameter tuning, or external validation. Although new reporting guidelines such as TRIPOD-AI and MINIMAR have been proposed to standardize disclosure and improve interpretability, adherence remains inconsistent within the surgical literature [119]. This lack of uniformity complicates comparisons across studies and impedes the ability of clinicians to critically evaluate the readiness of AI tools for clinical deployment.

Model overfitting is another recurrent concern. Because many cardiac surgery datasets include only a few thousand patients, the deep learning models, that are capable of encoding millions of parameters, are at risk of memorizing noise rather than learning clinically meaningful patterns. Overfitting can produce deceptively high internal performance that collapses upon external validation, a phenomenon well documented in AI applications within imaging, cardiology, and critical care [120,121]. The absence of rigorous external testing in many studies raises valid questions regarding the stability and reliability of the reported results (Table 4).

Even when performance is adequate, real-world integration remains challenging. High-performing algorithms often fail to improve patient outcomes when deployed at the bedside due to limited workflow compatibility, suboptimal user interfaces, alert fatigue, and insufficient clinician training. Early-warning systems in ICUs provide an instructive example: despite strong predictive accuracy, many have struggled to demonstrate clinical benefit because alerts did not prompt timely action or because clinical teams lacked structured response pathways [122]. For cardiac surgery, where care is highly protocolized and time-sensitive, integration barriers may be even more consequential.

Lastly, long-term outcome data and economic analyses are still nascent. While AI holds promise for improving survival, reducing complications, and lowering costs, few studies have rigorously evaluated these endpoints. Most cost-effectiveness analyses remain theoretical or rely on extrapolation rather than prospective measurement, leaving open questions regarding sustainability, resource utilization, and the economic value of AI-driven care pathways [123].

Taking everything into consideration, although artificial intelligence has demonstrated substantial promise across diagnostic, intraoperative, and postoperative domains, the current evidence base is constrained by methodological limitations, variable data quality, incomplete validation, and limited clinical integration. Addressing these gaps will require multicenter collaboration, standardized reporting, prospective trials, and thoughtful implementation frameworks. Only through such coordinated efforts can AI transition from experimental innovation to a dependable, widely adopted tool in adult cardiac surgery.

### 8.2. Mixed and Negative Experiences with Real-World AI Deployment

Although much of the published literature emphasizes the promise of artificial intelligence in cardiovascular medicine and surgery, real-world experience has revealed several examples of mixed, neutral, or unsuccessful implementation. These experiences provide critical insight into the barriers that limit translation from algorithmic performance to clinical benefit.

One of the most frequently reported challenges involves early-warning systems with high predictive accuracy but limited clinical impact. Multiple ICU-based AI alerting tools have demonstrated strong discrimination for outcomes such as sepsis, hypotension, or clinical deterioration, yet failed to improve patient outcomes after deployment. Post-implementation analyses often revealed alert fatigue, poor alignment with clinical workflows, or lack of predefined response protocols, resulting in delayed or inconsistent clinician action despite accurate predictions [122].

External validation failures represent another common limitation. AI models trained on single-center or homogeneous datasets frequently experience substantial performance degradation when applied to new institutions with different patient populations, perfusion strategies, anesthetic practices, or electronic health record structures. In cardiac surgery, such variability can profoundly affect outcomes such as vasoplegia, low-cardiac-output syndrome, and acute kidney injury, leading to models that internalize center-specific practice patterns rather than generalizable physiologic signals [115,116,118].

Several AI tools have also encountered user-interface and trust barriers. Clinicians may disregard algorithmic recommendations when outputs are poorly explained, conflict with clinical intuition, or lack transparency regarding contributing variables. Studies evaluating explainable AI interfaces suggest that opaque “black-box” predictions, even when accurate, are less likely to influence decision-making than models offering interpretable rationales.

Importantly, negative or neutral experiences do not imply failure of AI as a concept, but rather underscore the complexity of deploying predictive tools in high-stakes surgical environments. These lessons highlight that successful AI implementation depends as much on workflow integration, governance, and clinician engagement as on model performance itself. Recognizing and learning from unsuccessful deployments is therefore essential to designing AI systems that achieve durable, real-world impact in cardiac surgery.

## 9. Future Directions in Cardiac Surgical Artificial Intelligence

Although artificial intelligence has already begun to influence diagnostic evaluation, surgical planning, intraoperative assistance, and postoperative care in adult cardiac surgery, the next decade promises far more transformative developments. These advances will be driven by multimodal data integration, autonomous systems, real-time digital modeling, and the evolution of fully connected operating-room ecosystems. Together, these innovations point toward a future in which cardiac surgery becomes increasingly precise, personalized, and anticipatory.

A major frontier involves the emergence of multimodal AI models that combine electrocardiography, imaging, hemodynamic waveforms, genomic and proteomic data, and unstructured clinical notes to create a unified patient representation. Such foundation models, analogous to large language models but trained on medical signals and imaging, are expected to dramatically improve risk prediction, early-detection capabilities, and automated interpretation of complex physiologic patterns [124]. Early prototypes in cardiology have shown impressive performance across diverse tasks, raising the possibility that a single multimodal architecture could support preoperative risk stratification, intraoperative guidance, and postoperative prognostication.

**Figure 3:**
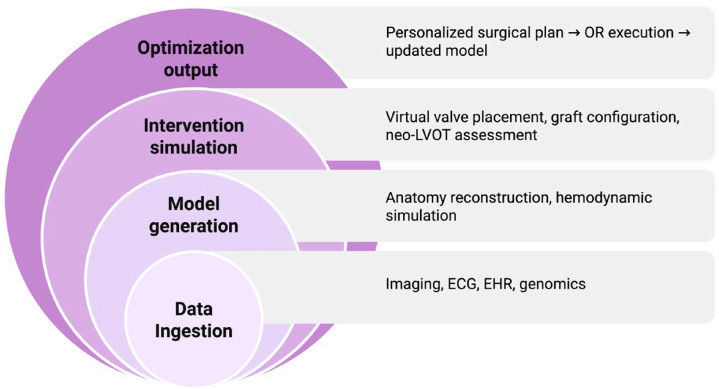
Digital Twin Workflow for Cardiac Surgery.

Another area of rapid development is the creation of digital twins, virtual replicas of individual patients that simulate cardiac anatomy, physiology, tissue properties, and hemodynamics (Figure 3). Digital twins integrate imaging, echocardiography, computational fluid dynamics, and machine learning to model potential outcomes of surgical or transcatheter interventions. In early feasibility studies, digital twins have been used to test valve deployment angles, assess graft flow distribution, and simulate the hemodynamic effects of complex aortic repairs [125,126]. As these models become more accurate and computationally efficient, they may allow surgeons to rehearse operations in a patient-specific virtual environment, select optimal conduit configurations, and predict complications before entering the operating room.

The evolution of semi-autonomous and autonomous robotic systems represents another promising trajectory. While current platforms remain largely teleoperated, advances in computer vision, force-sensing algorithms, and reinforcement learning suggest that autonomous performance of discrete surgical subtasks, such as suture placement, vessel probing, anastomotic alignment, or valve debridement, may become increasingly feasible [127]. The integration of real-time AI-driven anatomic recognition could enable robots to navigate tissue planes with minimal human adjustment, functioning as highly reliable procedural assistants. Although full autonomy remains distant in cardiac surgery due to its complexity and risk profile, meaningful partial autonomy may emerge first in structured, repetitive tasks within minimally invasive procedures.

AI is also poised to transform the hybrid operating room into a dynamic, learning environment. Next-generation OR platforms will likely feature continuous data exchange between imaging systems, perfusion consoles, anesthesia monitors, and robotic tools. These systems could generate real-time 3D reconstructions of operative anatomy, predict physiologic transitions minutes in advance, and provide proactive alerts regarding tissue stress, cannulation risk, or graft kinking [128]. Such developments may reduce human-factor error, enhance situational awareness, and allow for more standardized, reproducible performance of complex cardiac procedures.

Another important direction involves the expansion of real-time surgical video analytics. Computer-vision systems are rapidly advancing from detecting simple landmarks to segmenting tissue layers, quantifying bleeding, analyzing instrument trajectories, and identifying deviations from ideal technique. Within the next decade, AI is expected to provide continuous intraoperative skill assessment and automated coaching, helping surgeons refine their technique and maintain high performance throughout long or difficult procedures [129]. These tools may also serve as quality-assurance mechanisms, offering objective metrics for accreditation, training, and postoperative debriefing.

In parallel, significant progress is anticipated in precision postoperative care. Rich physiologic data streams from ICU monitors, imaging exams, lab results, and wearable sensors will increasingly be analyzed by predictive models capable of identifying early signs of deterioration well before clinical recognition. AI-enabled closed-loop systems for vasopressor titration, fluid management, ventilator control, and temperature regulation are already being tested and may eventually provide consistent, individualized hemodynamic optimization [130,131]. Remote monitoring systems equipped with AI-driven analytics will likely extend postoperative surveillance beyond hospital discharge, reducing readmissions and improving long-term outcomes.

Another promising domain is genomic, transcriptomic, and multi-omic integration. As large-scale biobank data grow, AI models may begin to link molecular signatures with surgical risk, graft durability, valve degeneration, and aortic aneurysm progression [132]. These insights could inform patient selection, guide operative strategy, and enable targeted perioperative therapies aimed at modulating mitochondrial dysfunction, inflammation, or extracellular-matrix remodeling.

The future also requires careful attention to ethical governance and regulatory frameworks. As AI models become more adaptive, continually learning from new data, regulatory agencies will need robust mechanisms for monitoring algorithmic drift, ensuring fairness, and protecting patient privacy. Advances in federated learning, differential privacy, and secure multiparty computation may enable large-scale model training across international centers without compromising data security [133,134].

Finally, achieving the full potential of AI in cardiac surgery will depend on the creation of global, harmonized data infrastructures. Collaborative networks that include diverse populations, standardized variable definitions, and high-frequency physiologic data are essential for building generalizable models that perform reliably across institutions. Several international initiatives have already called for multi-institutional surgical AI registries, which would enable continuous benchmarking, transparent model updating, and rigorous prospective validation [135,136,137].

Taken together, these innovations suggest that AI will fundamentally reshape nearly every phase of adult cardiac surgery. With the continued convergence of digital twins, multimodal learning, hybrid OR ecosystems, autonomous robotics, and precision perioperative care, the next decade may mark the transition from AI-enabled decision support to truly predictive, personalized, and simulation-driven surgical practice.

## 10. Conclusions

Artificial intelligence is rapidly reshaping the landscape of adult cardiac surgery, permeating each phase of the perioperative pathway—from diagnostic evaluation and risk stratification to surgical planning, intraoperative guidance, and postoperative critical care. What began as modest experiments in pattern recognition and image segmentation has evolved into a robust ecosystem of predictive analytics, computer-vision tools, multimodal learning systems, and AI-enabled robotic platforms. Together, these technologies are beginning to augment surgical precision, enhance situational awareness, and enable more personalized decision-making. Yet, the transformative potential of AI in cardiac surgery extends beyond technical innovation alone. By integrating continuously streamed physiologic data, rich imaging modalities, and longitudinal clinical information, AI offers a perspective that is more anticipatory than reactive—one capable of recognizing subtle physiologic signatures before deterioration becomes clinically apparent. Similarly, AI-driven simulations, digital twins, and hybrid operating-room ecosystems promise unprecedented opportunities for procedural rehearsal, real-time anatomic guidance, and individualized operative strategy. At the same time, significant challenges remain. The current evidence base is still dominated by retrospective analyses, with limited prospective or randomized evaluation. Issues of data quality, algorithmic bias, model transparency, regulatory oversight, and real-world workflow integration must be addressed before AI can reliably influence high-stakes surgical care. The field must move toward multicenter collaboration, standardized reporting frameworks, and robust governance structures to ensure safe and equitable adoption.

Looking ahead, the next decade is likely to mark a transition from discrete AI applications to fully integrated, learning-health-system architectures in which cardiac surgery is supported by continuous data exchange across the preoperative, intraoperative, and postoperative continuum. As multimodal foundation models, autonomous robotic functions, and precision perioperative pathways mature, AI will increasingly serve not as a tool but as a clinical partner, one that enhances, rather than replaces, the expertise of the cardiac surgical team. Ultimately, the promise of AI in adult cardiac surgery lies not only in improved efficiency or technical innovation, but in its potential to deliver more accurate diagnoses, safer operations, and better outcomes for patients. With thoughtful validation, ethical stewardship, and clinician-led implementation, artificial intelligence may become a cornerstone of next-generation cardiac surgical practice, ushering in an era of truly individualized, data-driven, and future-ready care.

## Figures and Tables

**Table 1:** Present and Potential AI Applications Across the Cardiac Surgical Perioperative Pathway.

Perioperative Phase	AI Application	Clinical Purpose	Representative Examples
Preoperative Diagnostics	Deep-learning ECG analysis	Detect subclinical LV dysfunction; predict long-term survival and operative risk	AI-ECG for EF < 35%; AI-derived cardiac age
	Automated CT/MRI segmentation	Rapid extraction of anatomy, calcification burden, chamber volumes	TAVR annulus sizing; aortic morphology modeling
	Echocardiographic video analysis	Predict RV failure, identify cardiomyopathies	Stanford LVAD RV-failure prediction model
	NLP extraction of risk variables	Automated identification of clinical features and comorbidities	EHR-driven early-warning systems
Risk Stratification	Machine-learning mortality/morbidity prediction	Improve upon STS and EuroSCORE II performance	ANN models; gradient-boosted trees; XGBoost risk scores
	Multimodal prognostic models	Integrate imaging, labs, ECG, and clinical notes for individualized risk	Foundation models using multimodal data
Surgical Planning/Structural Heart	Virtual valve implantation & neo-LVOT simulation	Prevent LVOT obstruction; optimize TMVR	Deep-learning TMVR simulation tools
	AI-driven TAVR planning	Valve sizing, PVL prediction, conduction disturbance risk	Automated CT analysis; ML-based sizing
	3D printing + ML segmentation	Preoperative rehearsal and hazard identification	Minnesota 3D cardiac models
Intraoperative Assistance	AI-guided hemodynamics (HPI)	Predict hypotension; optimize perfusion	HPI in cardiac ERAS pathways
	Computer vision in live video	Detect bleeding, track instruments, identify tissue planes	Blood-pixel CV models; OR Black Box
	Intelligent robotic functions	Tremor filtering, tool tracking, semi-autonomous suturing	STAR robot; AI-augmented robotics
Postoperative Care/ICU	Early-warning models	Predict AKI, LCOS, respiratory failure, delirium	eCART, AI AKI models, waveform analytics
	Closed-loop physiologic control	Automated vasopressor, ventilation, temperature adjustments	Reinforcement-learning ventilator control
	Remote AI monitoring	-detected arrhythmias, effusions, hemodynamic instability post-discharge	AI-enabled wearables and home telemetry

AI: Artificial intelligence; ECG: electrocardiogram; LV: left ventricular; EF: ejection fraction; TAVR: transcatheter aortic valve replacement; RV: Right Ventricle; LVAD: left ventricular assist device; NLP: natural language processing; EHR: electronic health record; STS: The Society of Thoracic Surgeons; ANN: Artificial Neural Networks; LVOT: left ventricular outflow tract; TMVR: transcatheter mitral valve replacement; PVL: paravalvular leak; ML: machine learning; HPI: Hypotension Prediction Index; ERAS: Enhanced Recovery After Surgery; CV: Computer Vision; OR: Operating Room; AKI: acute kidney injury; LCOS: low-cardiac-output syndrome; eCART: electronic Cardiac Arrest Risk Triage.

**Table 2:** Real-World Evidence and Outcome Metrics of AI Applications in Adult Cardiac Surgery.

Perioperative Phase	AI Application	Model Type	Study Design/Evidence Level	Sample Size	Performance Metrics	Clinical Outcome Impact	Comparator	Deployment Status
Preoperative	AI-enabled ECG for LV dysfunction	CNN	Retrospective, external validation	>40,000	AUC ~0.85	Prediction of future LV dysfunction	Cardiologist ECG interpretation	Retrospective
Preoperative	AI-ECG “cardiac age”	Deep learning	Retrospective cohort	13,808 CABG pts	HR ↑ with age gap	Worse 5- and 10-yr survival	Chronologic age	Retrospective
Preoperative	Echo-based RV failure prediction (LVAD)	CNN	Multicenter retrospective	941	AUC 0.73	Improved RV failure risk identification	Clinical scores; experts	Retrospective
Preoperative	ML mortality prediction	XGBoost/ANN	Registry-based retrospective	4874	AUC > STS	Improved mortality & morbidity prediction	STS risk calculator	Retrospective
Intraoperative	Hypotension Prediction Index (HPI)	ML waveform analysis	Prospective observational	>600 cardiac pts	Sens/Spec > 80%	↓ ventilation time; improved stability	Standard MAP monitoring	Implemented
Intraoperative	Autonomous suturing (STAR)	CV + ML	Preclinical	N/A	Technical accuracy metrics	Superior suture consistency	Expert surgeons	Experimental
Intraoperative	OR Black Box motion analysis	CV + ML	Prospective observational	Hundreds	Error detection > 90%	Identification of unsafe motions	Human observation	Pilot deployment
Postoperative	AKI prediction	ML/RNN	Retrospective	1000–10,000	AUC 0.75–0.85	Earlier AKI risk identification	Logistic regression	Retrospective
Postoperative	ICU early-warning systems	ML ensemble	Prospective & implemented	>50,000	AUC ~0.80	↓ ICU transfers; ↓ mortality	Rule-based alerts	Implemented
Postoperative	Remote AI monitoring	ML	Prospective pilot	Hundreds	Detection accuracy reported	↓ readmissions	Standard follow-up	Pilot

Abbreviations: AI, artificial intelligence; ANN, artificial neural network; AUC, area under the curve; CABG, coronary artery bypass grafting; CNN, convolutional neural network; CV, computer vision; ECG, electrocardiogram; ICU, intensive care unit; LV, left ventricle; LVAD, left ventricular assist device; MAP, mean arterial pressure; ML, machine learning; RNN, recurrent neural network; STS, Society of Thoracic Surgeons; RV: Right Ventricle; XGBoost: extreme gradient boosting; OR: Operating Room; AKI: acute kidney injury.

**Table 3:** AI Modalities and Their Clinical Contributions in Adult Cardiac Surgery.

AI Modality	Core Capabilities	Clinical Contributions	Key Domains in Cardiac Surgery
Machine Learning (ML)	Pattern recognition; structured data modeling	Improved risk stratification; complication prediction	STS augmentation, mortality/morbidity models, AKI prediction
Deep Learning (DL)	Image, waveform, and time-series analysis	Automated imaging interpretation; physiologic signature detection	Echocardiography, MRI, AI-ECG, perfusion waveforms
Computer Vision (CV)	Real-time surgical video interpretation	Intraoperative bleeding quantification; skill assessment	OR Black Box, robotic guidance, structural interventions
Natural Language Processing (NLP)	Extraction of free-text clinical data	Automated registry development; continuous monitoring	Operative notes, radiology reports, EHR extraction
Reinforcement Learning (RL)	Adaptive decision policies based on feedback	Closed-loop physiologic control; autonomous systems	Ventilation control, perfusion optimization
Foundation/Multimodal Models	Cross-domain integration of images, signals, genomics, and text	Holistic patient modeling; universal risk profiles	Digital twins, comprehensive perioperative prediction
Simulation & Digital Twins	Virtual physiological modeling	Preoperative rehearsal; device simulation	TMVR, aortic repair, TEVAR computational planning

STS: Society of Thoracic Surgeons; AKI: acute kidney injury; MRI: magnetic resonance imaging; AI: Artificial intelligence; ECG: electrocardiogram; OR: Operating Room; EHR: electronic health record; TMVR: transcatheter mitral valve replacement; TEVAR: thoracic endovascular aortic repair.

**Table 4:** Challenges and Barriers to AI Adoption in Cardiac Surgery.

Challenge Category	Description	Impact on Clinical Adoption	Examples
Data Quality & Generalizability	Single-center, retrospective datasets; inconsistent variable definitions	Reduced external validity; model degradation	Variability in LCOS and vasoplegia definitions
Transparency & Explainability	Black-box models with limited interpretability	Clinician distrust; regulatory resistance	Lack of rationale for AI risk predictions
Bias & Fairness	Underrepresentation of minority groups, women, older adults	Performance disparities; potential harm	Differential calibration of models by race/sex
Workflow Integration	Poor interface design; alert fatigue; limited EHR integration	Failure to improve outcomes despite strong predictive performance	Early-warning systems with low response rates
Regulatory & Liability Issues	Unclear oversight for adaptive algorithms	Hesitancy to deploy AI in high-risk procedures	FDA SaMD change-control challenges
Model Drift & Maintenance	Changing patient populations, surgical techniques	Performance decline without retraining	Aortic and valve practice evolution
Economic & Resource Constraints	Cost of adoption, staff training, IT infrastructure	Slower implementation; inequitable access	Hybrid OR integration costs; cloud computing fees

LCOS: low-cardiac-output syndrome; AI: Artificial intelligence; EHR: electronic health record; FDA: Food and Drug Administration; SaMD: software as a medical device; OR: Operating Room.

## Data Availability

No new data were created or analyzed in this study. Data sharing is not applicable to this article.
